# Essential newborn care practice and its associated factors in Southwest Ethiopia

**DOI:** 10.1186/s13690-021-00568-6

**Published:** 2021-03-31

**Authors:** Haimanot Abebe, Daniel Adane, Solomon Shitu

**Affiliations:** 1 Department of Public health in Reproductive Health, College of Medicine and Health Science, Wolkite University, Wolkite, Ethiopia; 2 Department of Midwifery, College of Medicine and Health Science, Wolkite University, Wolkite, Ethiopia

**Keywords:** Practice, Essential newborn care, Newborn, Gurage zone, Ethiopia

## Abstract

**Background:**

Essential newborn care is a wide-ranging strategy intended to improve the health of newborns by implementing appropriate interventions. Approximately in 2018, an estimated 2.5 million children died in their first month of life, which is approximately 7000, newborns every day, with about a third of all neonatal deaths occurring within the first day after birth. Even though the most cause of death is preventable the burden of neonatal death is a still high in developing countries including Ethiopia. Therefore this study is aimed to assess the level of essential newborn care practice among mothers who gave birth within the past six months in Gurage Zone, Southwest Ethiopia**.**

**Methods:**

A community-based cross-sectional study was conducted among mothers who gave birth within the past six months in Gurage Zone, Southwest Ethiopia. For the quantitative part, 624 study participants were involved by using a multi-stage sampling method. A systematic random sampling technique was to reach the study subjects. Data entry was carried out by Epi data version 4.0.0 and analysis was done by SPSS window version 24. Binary and multivariate logistic regressions were used to identify associated factors. For the qualitative part, three focus group discussions (FGD) with purposively selected 30 mothers were involved. The data were analyzed deductively by using the thematic framework analysis approach by using Open code version 4.02.

**Result:**

Overall good essential newborn care practice was found to be 41.0% [95%CI, 36.6–44.7]. Being urban residence [AOR 1.70, 95%CI: 1.03–2.79], attending antenatal care visit [AOR = 3.53, 95%CI: 2.14–5.83], attending pregnant mothers meeting [AOR = 1.86, 95%CI: 1.21–2.86], had immediate postnatal care [AOR = 3.92, 95% CI: 2.65–5.78], and having good knowledge about ENC [AOR = 2.13, 95% CI: 1.47–3.10] were significantly associated with good essential newborn care practice.

**Conclusion:**

This study indicated that the magnitude of essential newborn care practice was low. Thus, a primary health care provider should regularly provide ENC for newborns and take opportunities to counsel the mothers about ENC during pregnant mothers meeting and MCH services sessions.

**Supplementary Information:**

The online version contains supplementary material available at 10.1186/s13690-021-00568-6.

## Background

Globally, as mortality among children under five declined, deaths among these children are more and more concentrated in the first days of life. These makes focus on newborn care more critically than ever before [1]. In 2018, an estimated 2.5 million children died in their first month of life, which is approximately 7000, newborns every day, with about a third of all neonatal deaths occurring within the first day after birth, and close to three-quarters occurring within the first week of life [2, 3].

Sub-Saharan Africa had the highest neonatal mortality rate in 2019 at 27 deaths per 1000 live births, followed by Central and Southern Asia with 24 deaths per 1000 live births. A child born in sub-Saharan Africa or in Southern Asia is ten times more likely to die in the first month than a child born in a high-income country. The majority of neonatal mortality in developing countries are related to the conditions of labor, intrapartum, and poor immediate newborn care practices [4, 5]. More than 60% of infant and 40% of under-five deaths in Ethiopia are neonatal deaths [6]. Hence, the Ethiopian government will have huge work for curbing neonatal and child mortality.

To elaborate the, WHO has stated that, “a customary practice that reduces newborn morbidity and mortality has been identified as indispensable and these include essential newborn care.” Essential newborn care (ENC) is defined as a strategic approach planned to improve the health of new-born through interventions before, during, and after pregnancy, immediately after birth, and during the postnatal period [7, 8]. Enhancing neonatal survival begins with upgrading the health status of their mothers. It is a cost-effective intervention that improves both maternal and neonatal health as well as their nutritional status. Cord care, neonatal feeding, and thermal care are the recommended essential newborn care practice by mothers at home during the postnatal period for all new-born babies [7–9].

Essential newborn care (ENC) is a set of benchmark every neonate warrant regardless of where it is born. It is designed to protect the newborn from an adverse environmental condition [10–13]. Surveys conducted in Ethiopia indicated the prevalence of essential newborn care practice range from 11.7–65.1%. Women’s age, education, residency, economic status of women, health facility accessibility, occupation, parity, women knowledge on essential newborn care practice, counseling during the perinatal period, ANC attending and getting immediate postnatal care were among the factors associated with essential newborn care practice [14–19].

In Ethiopia, the health extension program is the most important community-based newborn care (CBNC) packaging that aims to improve newborn survival. This includes applying a newborn care package along with the continuum of care from pregnancy to post-partum period through frontline community workers, such as improving sepsis management [10]. Although the essential newborn practice is effective and has been widely promoted, data on women’s practice and it’s contributing factors towards essential newborn care in a study setting is limited. Therefore, this study is aimed to assess the essential newborn care practice of mothers and associated factors in Gurage Zone, Southwest Ethiopia.

## Methods

### Study setting, period, and design

A community-based mixed study was conducted in the Gurage Zone between March to May 2020. The Gurage zone is one of the administrative zones in South Ethiopia. It has 13 Woreda and two town administrations**.** Wolkite town is the capital of the Gurage zone. It is found 153 km southwest of Addis Ababa, the capital of Ethiopia. According to the 2007 national household census, the Gurage zone has a total population of 1,279,646, of which 657,568 are women [20]. There are seven hospitals (five public and two non-governmental) serving the total population in the zone. Five of the hospitals in the zone are primary hospitals, and the remaining two is a general zonal hospital. All hospitals deliver comprehensive emergency obstetric care services. Additionally, 72 health centers provide basic emergency obstetric care services in the Gurage zone.

### Populations

All mothers who gave birth in six months before the study period constituted the source population. Mothers who were found in selected Keble (small administrative unit in Ethiopia) comprised the study population for this study.

### Eligibility criteria

All mothers who gave birth in the past six months before the study period and were residents for at least 6 months in the study area was included in this study. Those mothers who did not able to communicate with the interviewer, seriously ill, and mothers who delivered a baby died before the data collection period were excluded.

### Sample size determination

The sample size for the study was calculated using Epi Info™ version 7 StatCalc function of sample size calculation for population survey at 95% confidence interval (CI), 5% margin of error, considering 44.1% of mothers had good essential newborn care practice from the related study in Nekemte city, West Ethiopia [12] and adding 10% non-response rate, a total of 416 study participants were estimated for this study. However, due to the design effect, the final sample used for this study was 624. The design effect of 1.5 was used to calculate the sample sizes. For the qualitative part of the study, three focus group discussions (FGD) with ten mothers in each group were involved depending on idea saturation.

### Sampling procedure

For quantitative data, a multi-stage sampling method was used. Purposive sampling was used for the qualitative data. From the woreda of the zone, five woreda and one town administration were selected by a simple random sampling technique using the lottery method. Then, three kebeles from each woreda and two kebeles from Butajira town were randomly selected. Households with mothers who gave birth in the past six months before the study period were listed out from the family folder of health extension workers (HEW) and the study participants were recruited by using a systematic random sampling technique.

### Data collection tools and procedure

A pre-tested, structured questioner was administered to collect quantitative data. An interview control for discussion was accustomed to collect qualitative information. The tool was developed after exhaustively reviewing different relevant kinds of literature [14–19]. The questionnaire comprises socioeconomic characteristics, information on maternal and child health service, mothers’ knowledge on newborn care, and neonatal danger signs. The data were collected by trained 12 diploma nurses and supervised by three BSc holder nurses who were fluent in the local language. The data collectors possessed the information by face to face interview of mothers at the household level. To generate qualitative information, an FGD was conducted. Each meeting of the FGD was tape-recorded after obtaining written and signed voluntary consent from the FGD participants. Those participants were selected by the principal investigator and the discussion was conducted until saturation of ideas occurred within the group. The discussion was moderated by the principal investigator and one other assistant (data collector) took notes and recorded all the information of the FGD.

### Operational definitions

#### Essential newborn care practice

The practice was reported as ‘good’ for mothers who practiced three components (safe cord care, optimal thermal care, and good neonatal feeding) appropriately while the practice was reported ‘poor’ if at least one component was missed from three components.

#### Safe cord care

Defined as keeping the cord, clean and dry without application of any substance on the cord stump except medically indicated medications like chlorhexidine.

#### Optimum thermal care

A new-born wrapped in a clean, and dry cloth and delay bathing a new-born delivery for 24 h to prevent hypothermia.

#### Neonatal feeding

Defined as initiating breastfeeding within the first one hour after birth, giving no pre-lacteal, and feeding the child with colostrum.

#### Knowledge of essential new-born care

Knowledge was ‘good’ for mothers who responded greater than or equal to the mean value of knowledge-related questions correctly whereas knowledge was ‘poor’ for mothers who responded less than the mean value of knowledge-related questions.

### Data quality control

To ensure quality, the questionnaire was initially drafted in the English language and then translated into the local language, *Gistane* by verified translators. Uttermost, before data collection, the questionnaire was back-translated into English to cinch precision. Questionnaires were pre-tested. Data were checked for completeness, accuracy, clarity, and consistency before being entered into the software. Proper coding and categorization of data were maintained for the quality of the data to be analyzed. Double data entry was used to ensure validity and compare to the original data.

### Data analysis and processing

The quantitative data were coded, cleaned, edited, and entered into Epi data version 4.0.0, then exported to SPSS window version 24 for analysis. Binary logistic regression was used to assess the association between each independent variable and outcome variable. Model fitness tests were checked using a Hosmer–Lemeshow goodness-of-fit and Omnibus tests.

All variables with *P <* 0.25 in the bivariate analysis were included in the final model of multivariate analysis to control all possible confounders. Besides, variables that were significant in previous studies and from a context point of view were included in the final model even if the above criteria were not meet. A variance inflation factor *>* 10 and standard error *>* 2 were considered as suggesting the existence of multi co-linearity.

*P* value lees than 0.05 with 95% confidence level were used to give out statistical significance. The focus group discussion audios were initially transcribed verbatim in the local language, *Gistane*, and then translated into English transcripts by the principal investigator. Data were analyzed deductively by using a thematic framework analysis approach and qualitative data analysis software Open Code version 4.02 was used. Each transcript was gingerly screened and coded.

## Results

### Socio-demographic characteristics

In this study, 608 participants responded to the questionnaire with a total responses rate of 97.4%. The majority of the respondents were in the age group 25–34 and the mean age of study participants were 27.38 (±4.81 SD). Of the respondents, the majority were Gurage by ethnicity 447 (73.5%) and lived in rural locations constitutes 502(82.6%). Orthodox Christianity was the dominant religion of 380 (62.5%) among study participants (See Table 1).

**Table 1:** Socio-demographic characteristics of study participants in Gurage zone, Southwest Ethiopia, 2020 (*n* = 608)

Variable	Frequency	Percent
**Age (Year)**		
15–24	115	27.8
25–34	242	58.6
≥ 35	56	13.6
**Ethnicity**		
Gurage	447	73.5
Amhara	123	20.2
Oromo	38	6.3
**Religion**		
Orthodox	380	62.5
Muslim	172	28.3
Protestant	56	9.2
**Residence**		
Rural	502	82.6
Urban	106	17.4
**Education**		
No formal education	210	34.5
Primary Education	233	38.3
Secondary and above	165	27.2
**Occupation**		
House wife	398	65.5
Merchant	81	13.3
Government Employer	111	18.2
Daily laborer	18	3.0
**Head of Household**		
Mother	484	79.6
Husband	124	20.4
**Average monthly income**		
≤ 1999	175	28.8
2000–3999	288	47.4
≥ 4000	145	23.8

### Maternal and child health services

Of the respondents, 472 (77.6%) had ANC follow-up. Two hundred two (42.8%) had one to three visits, 234 (49.6%) had four visits and 36 (7.6%) had five and more visits. During ANC follow-up the majority of 322 (53.0%) were advised about ENC. Three hundred sixty-four (59.9%) had delivered at a health institution and 354 (58.2%) had immediate postnatal care. The majority of the neonates 344 (56.6%) were male by sex and 516 (84.9%) mothers did not face any complications during delivery.

Three hundred eighty-seven (63.7%) of the study participants were assisted by a family member during the postnatal period, 113 (18.6%) of the study participants were assisted by a mother during the postnatal period, 73(12.0%) of the study participants were assisted by a neighbor during the postnatal period, and 35(5.8%) of the study participants were assisted by HEW during the postnatal period. Three hundred sixty-seven (60.4%) of the participants had given two up to four childbirth, 140 (23.0%) of the participants had given more than five childbirth and 101 (16.6%) of the participants had given one childbirth. Of the respondents, 329 (54.1%) had planned pregnancy during the last pregnancy (See Table 2).

**Table 2:** Maternal and child health services among study participants in Gurage zone, Southwest Ethiopia, 2020 (*n* = 608)

Variable	Frequency	Percent
**ANC follow-up**		
Yes	472	77.6
No	136	22.4
**Attended monthly pregnant mothers meeting**		
Yes	322	53.0
No	286	47.0
**Place of delivery**		
Home	244	40.1
Health institution	364	59.9
**Immediate post-natal care**		
Yes	354	58.2
No	254	41.8
**Faced complication during pregnancy**		
Yes	92	15.1
No	516	84.9
**Care giver during postnatal period**		
Family	387	63.7
Mother	113	18.6
Neighbor	73	12.0
HEW	35	5.8
**Planned pregnancy**		
Yes	329	54.1
No	279	45.9
**Party**		
1	101	16.6
2–4	367	60.4
≥ 5	140	23.0

### Knowledge of participants about essential newborn care and neonatal danger signs

Regarding, to neonatal danger signs knowledge of participants, 294(48.4%) of them had good knowledge [who are mentioned greater than or equal to the mean value]. Furthermore, 293(48.2%) mentioned poor sucking and 387 (63.7%) mentioned fever, 234 (38.5%) mentioned difficulty of breathing, 69 (11.3%) mentioned comma, 121 (19.9%) mentioned grunt, 86 (14.1%) mentioned hypothermia, 79 (13.0%) mentioned omphalitis, 160 (26.3%) mentioned umbilical infection, 118 (19.4%) mentioned jaundice and 119 (19.6%) mentioned vomiting.

**Fig. 1:**
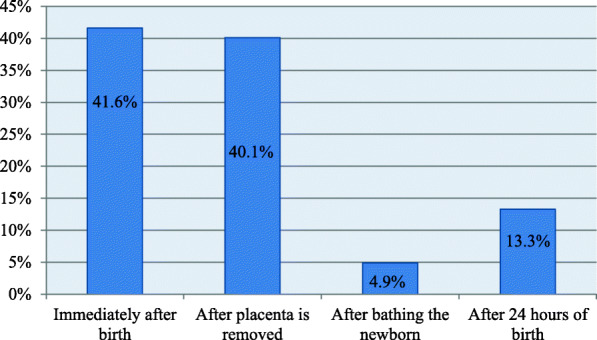
Breastfeeding initiated time among mothers in Gurage zone, Southwest Ethiopia, 2020 (*n* = 608)

Concerning essential newborn care knowledge, 304(50.0%) of the participants had good knowledge (responded greater than the mean value of knowledge related questions). Study participants were asked about breastfeeding initiation time, bathing time, and knowledge about neonatal danger signs. Out of study participants, 253 (41.6%) stated that breastfeeding initiation time was within one hour (See Fig. 1).

**Fig. 2:**
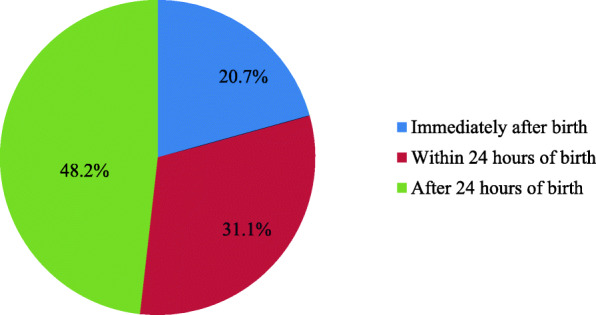
Time of bathing among mothers in Gurage zone, Southwest Ethiopia, 2020 (n = 608)

Two-hundred ninety-three (48.2%) of the study participants stated that the newborn had been bathed after 24 h post-delivery (See Fig. 2).

### Essential newborn care practice

In this study, 249(41.0%) of mothers had a good practice on ENC**.** Of the respondents, 489 (80.4%) applied substances on the stump, 276 (56.5%) applied ointment/ powder, 181(37.0%) applied butter, 18 (3.7%) applied animal dung and 14 (2.8%) applied ash on the stump. The qualitative finding was also supplemented the quantitative one in that still there are problems in the coverage of some of the essential newborn care practice. The qualitative part particularly focused on the cord are, thermal care, and breastfeeding.

According to the opinions of most of the respondents “*The common practice reported by the discussants in the qualitative findings was the application of fresh butter on and around the stump, whether delivery occurs at home or health facility. A 19-year-old FGD discussant noted that “I delivered my first newborn three months back at home by the help of my mother. After delivery, my mother tied the cord with a thread and cut it with a new blade that I was bought from the market. To cut the cord, my mother measure from the babies’ abdomen with her fingers. If the cord was tied and cut after one finger from the abdomen of the newborn it can be dropped within one day and if tied and cut after two fingers from the abdomen of the newborn it can be dropped within two days*.. ... .*. During the first 7 days after delivery, I was applied fresh butter on the stump to prevent dryness and to make it soft*...”

Colostrum was given for 544 (89.5%) of the babies of respondents after delivery and pre-lacteal feeding was given for 65 (10.7%) of the babies.

Among those who gave pre-lacteal feeding 35 (53.8%) of the mothers gave plain water, 16 (24.6%) gave animal milk, 12 (18.5%) gave butter and 2 (3.1%) gave honey. *A 34-year old FGD discussant mother said that “I had no antenatal care follow-up. And I was delivered at home with the help of traditional birth assistant. After delivery the attendants gave me Keneto and Tella (traditional beverage) before starting breastfeeding, they consider this would be enhanced mother breast milk and help newborns to initiate breastfeeding. Then the assistant informed me to stay for some time to breastfeed the neonate.*

Four hundred sixteen (68.4%) of the newborn babies had wiped and 491 (80.8%) had wrapped the baby in cloth after delivery. Of the participants, 396(65.1%) were used to warm water at the time of bath, and 518(85.2%) had used a new cloth to wrap.

*A 30 years old FGD discussant said: "I gave birth to my child four months back with the help of my mother. As soon as, the placenta was escape, she bathe me and my newborn baby with cold water. As to me, this is what all women in our community practice...." the discussant added, both I and my newborn is contaminated with dirty blood. That is why we used immediate showering (washing)....."*

Four hundred nineteen (68.9%), of the participants, were used as a boiled instrument to cut the cord. Various instruments were used to cut the cord, with 376 (61.8%) respondents stating they used a new string/thread, 139 (22.9%) stating they cord tie, and 10 (1.6%) stated cord not tied.

*Most of the discussants in the qualitative sentence explained that the cord was tied by thread or string and cut by a new blade if delivery occurs at home. Previously the cord was also tied apart from the Enset (Traditional plant in Ethiopia). But, the society renowned they are moving distant from this trend as improved awareness of the risks has been created by health extension workers (See*

**Table 3:** Mothers practice on essential newborn care in Gurage zone, Southwest Ethiopia, 2020 (*n* = 608)

Variable	Frequency	Percent
**Temperature of water used to bath**		
Cold	212	34.9
Warm	396	65.1
**Wiped within ten minute after delivery**		
Yes	416	68.4
No	192	31.6
**Wrapped by clothe after delivery**		
Yes	491	80.8
No	117	19.2
**Type of cloth used to wrap**		
New cloth	518	85.2
Clean and dry old cloth	62	10.2
Solid and old cloth	32	4.6
**Instrument boiled**		
Yes	419	68.9
No	189	31.1
**Applied substances on the stump**		
Yes	489	80.4
No	119	19.6
**Cord tie**		
New string/thread	376	61.8
Cord tie	139	22.9
Cord not tied	93	15.3
**Gave colostrum**		
Yes	544	89.5
No	64	10.5
**Pre-lacteal feeding**		
Yes	65	10.7
No	543	89.3

table 3*)*

### Factors associated with essential new-born care practices

In this study, being an urban resident, attending antenatal care visits, getting immediate postnatal care visits, attending pregnant mothers’ meetings, and having good knowledge about ENC were factors significantly associated with good essential newborn care practices.

Mothers who had urban residency were found to have a statistically significant association with essential new-born care practices. Those who had urban residency were 1.70 [AOR 1.70, 95%CI: 1.03–2.79] times more likely to practice essential new-born care as compared with those women who had a rural residency.

Mothers who had attended antenatal care visit were 3.53 times and who had attending pregnant mothers meeting were 1.86 times more likely to practice good essential newborn care, (AOR = 3.53, 95%CI: 2.14–5.83) and (AOR = 1.86, 95%CI: 1.21–2.86) respectively. The odds of good ENC practice were 3.92 among mothers who had immediate postnatal care and 2.13 among mothers who had good knowledge about ENC, (AOR = 3.92, 95% CI: 2.65–5.78) and (AOR = 2.13, 95% CI: 1.47–3.10) respectively (See Table 4).

**Table 4:** Factors associated with essential new-born care practices in Gurage zone, Southwest Ethiopia, 2020 (*n* = 608)

Variables	Essential newborn care practice		(95% CI)	
	Good	Poor	Crude OR	Adjusted OR
**Educational status**				
Secondary education and above	74 (29.7%)	91 (25.3%)	1.46 (0.97–2.22)	1.06 (0.62–1.82)
Primary education	100 (40.2%)	133 (37.1%)	1.35 (0.92–1.99)	0.92 (0.59–1.43)
No formal education	75 (30.1%)	135 (37.6%)	1.00	1.00
**Residences**				
Urban	50 (20.1%)	56 (15.6%)	1.36 (0.89–2.07)	**1.70 (1.03–2.79)**^**^**^
Rural	199 (79.9%)	303 (84.4%)	1.00	1.00
**Occupation**				
Employed	52 (20.9%)	59 (16.4%)	1.34 (0.88–2.03)	0.81 (0.47–1.40)
Unemployed	197 (79.1%)	300 (83.6%)	1.00	1.00
**Party**				
≥ 5	62 (24.9%)	78 (21.7%)	1.49 (0.88–2.54)	1.42 (0.77–2.61)
2–4	152 (61.0%)	215 (59.9%)	1.33 (0.84–2.11)	1.25 (0.72–2.12)
1	35 (14.1%)	66 (18.4%)	1.00	1.00
**Pregnancy planned**				
Yes	142 (57.0%)	187 (52.1%)	1.22 (0.88–1.69)	0.91 (0.59–1.39)
No	107 (43.0%)	172 (47.9%)	1.00	1.00
**ANC follow up**				
Yes	222 (89.2%)	250 (69.6%)	3.56 (2.57–5.67)	**3.53 (2.14–5.83)**^**^^**^
No	27 (10.8%)	109 (30.4%)	1.00	1.00
**Immediate PNC**				
Yes	194 (77.9%)	160 (44.6%)	4.39 (3.05–6.32%)	**3.92 (2.65–5.78)**^**^^**^
No	55 (22.1%)	199 (55.4%)	1.00	1.00
**Place of delivery**				
Health facility	142 (57.0%)	222 (61.8%)	0.82 (0.59–1.14)	1.02 (0.69–1.49)
Home	107 (43.0%)	137 (38.2%)	1.00	1.00
**Attending pregnant mothers meeting**				
Yes	152 (61.0%)	170 (47.4%)	1.74 (1.25–2.49)	**1.86 (1.21–2.86)^^^**
No	97 (39.0%)	189 (52.6%)	1.00	1.00
**Knowledge of mother**				
Yes	157 (63.1%)	147 (40.9%)	2.46 (1.77–3.43)	**2.13 (1.47–3.10)**^**^^**^
No	92 (36.9%)	212 (59.1%)	1.00	1.00

***^****Significant with P = 0.037,* ***^^****Significant with P < 0.001 and* ***^^^****Significant with P = 0.004*

## Discussion

The overall good essential newborn care practice was found to be 41.0% [95%CI, 36.6–44.7]. This finding is quite low compared to what it should be. The result is vital for healthcare planners. Hence, this knowledge can be used to build relevant programs, channeling scarce resources for teaching what is needed as opposed to imparting messages that are already known.

Overall, the magnitude of essential new-born care practice was 41.0%. This is nearly similar to a study conducted in Chencha district of Ethiopia (38.4%) and Nekemte city, western Ethiopia (44.1%) [11, 19]. This study was higher than a study conducted higher than studies conducted in Awabel District, Amhara region of Ethiopia (23.1%) and Eastern Uganda (11.7%) [12, 21]. But, lower than two studies done in South West Ethiopia (59.5%) and Eastern Tigray of Ethiopia (92.9%) [14, 22]. The discrepancy of these findings might be attributed to the difference in methods used and study settings, sociodemographic characteristics of the study participants, and availability and accessibility of health service infrastructures.

Attending ANC visit, attending pregnant mother meeting, knowledge of the mothers about essential new-born care practices, getting immediate postnatal care, and having urban residence were factors associated with essential new-born care practices. Those women who had accessed to ANC visits were 3.53 times more likely to practice essential new-born care as compared with those women who didn’t visit ANC at all for the current delivery. This finding is consistent with studies conducted in the Nekemte, and Chencha districts of Ethiopia [11, 19]. The possible reason could be those mothers who visited ANC would get counseling about health prevention and promotion which is believed to increase knowledge and practice of mothers about essential newborn care [23]. The odds of practice were 1.86 among mothers who attended pregnant mothers’ meetings. This is in line with the study done in Awabel District, Amhara Region, and Chencha district Southern, Region of Ethiopia [11, 12]. Mothers who had immediate postnatal care were 3.92 times and good knowledge about essential newborn care was 2.13 times more likely to practice essential newborn care. This is in line with studies done in Chencha southern Ethiopia, Awabel District, Amhara Region of Ethiopia, Nekemte western Ethiopia Nepal, and Bachauli and Khairahani [11, 12, 19, 24, 25]. The explanation for this is mothers who had PNC discussed and were counseled about essential newborn care with health extension workers or other health care providers and mothers who had good knowledge about essential newborn care are more likely to practice essential newborn care.

Regarding, the association of residency with essential newborn care practice in this study, those who have urban residency were nearly 1.70 times more likely to practice essential newborn care than those who have rural residency. This is in-line with the fact that women that are urban residence will have information that could assist them in making decisions regarding healthy behaviors including maternal and child health education, and promotion specifically essential newborn care practice**.** Hence, women who have urban residency will have access and availability of infrastructures like mass media and others that could enable them to be aware of the benefit of ENCP. The findings of the study are consistent with the study findings from Northwest and Southeast Ethiopia [26–28].

## Conclusion

This study indicated that the magnitude of essential newborn care practice was low. In nutshell, strengthen awareness creation activities on ENC by disseminating health information and developing communication strategies to promote positive behaviors both at the facility and community level. The primary health care provider should regularly provide ENC for newborns and take opportunities to counsel the mothers about ENC during pregnant mothers meeting and MCH services sessions are recommended.

## Supplementary Information

**Additional file 1.** English Version Questionnaire.

## Data Availability

The full data set and other materials about this study can be obtained from the corresponding author on reasonable requests.

## Abbreviations

ANC: Antenatal Care
ENCP: Essential Newborn Care Practice
MCH: Maternal and Child Health
PNC: Postnatal Care
SPSS: Statistical package for social science
