# Supplementary material for: Essential newborn care practice and its associated factors in Southwest Ethiopia
Source: Arch Public Health. 2021 Mar 31;79:42. doi: 10.1186/s13690-021-00568-6 (PMC8011223; doi:10.1186/s13690-021-00568-6)
Supplement: Supplementary file 1 — Additional file 1. English Version Questionnaire. [file 13690_2021_568_MOESM1_ESM.pdf]

## English Version Questionnaire

**Interview Record for Quantitative Data** **Identification Number** [ ]

Questionnaire developed for essential newborn care practice and its factors among mothers who gave birth within the past six months in Gurage Zone, Southern Ethiopia, 2020

| S.No. | Question | Response | Skip |
|---|---|---|---|
| **Part I: Socio- demographic Characteristics** | | | |
| 101 | Age of the mother | /_____________/ in complete year | |
| 102 | Ethnicity | 1. Gurage<br>2. Amhara<br>3. Oromo<br>4. Other, Specify_____________ | |
| 103 | Educational status of mother | 1. Cannot able to read and write<br>2. Can read and write<br>3. Grade 1-8<br>4. Grade 9-12<br>5. College and above | |
| 104 | Religion | 1. Orthodox<br>2. Catholic<br>3. Protestant<br>4. Other, Specify_____________ | |
| 105 | Occupation of mother | 1. House wife<br>2. Merchant<br>3. Government employer<br>4. Daily laborer<br>5. Other, Specify______________ | |
| 106 | Place of residence | 1. Urban<br>2. Rural | |
| 107 | Head of Household | 1. Mother<br>2. Husband | |
| 108 | Average monthly income | /________________/ in ETH birr | |
| **Part II: Maternal and Child Health Services** | | | |
| 201 | Do you have ante natal care visit? | 1. Yes<br>2. No | If "No" Skip to Q 203 |
| 202 | How many times you received antenatal care service from health care provider? | _________________(in number) | |
| 203 | Was your pregnancy planned | 1. Yes<br>2. No | |
| 204 | Have you attended monthly | 3. Yes | |

| | | | |
|---|---|---|---|
| | pregnant mother's group meeting? | 4. No | |
| 205 | Where you gave birth? | 1. Health center<br>2. Hospital<br>3. Health post<br>4. Home | |
| 206 | Do you get assistance during delivery from skilled birth attendant? | 1. Yes<br>2. No | If "No" Skip to Q 208 |
| 207 | Who gave delivery assistance for you? | 1. Health care provider<br>2. Family<br>3. Neighbor<br>4. Relatives(mother in law)<br>5. Traditional Birth Attendants (TBAs)<br>6. Health Extension Workers (HEWs)<br>7. Other, Specify______________ | |
| 208 | Had you faced any type of complication during the delivery? | 1. Yes<br>2. No | |
| 209 | Sex of your child? | 1. Male<br>2. Female | |
| 210 | Do you have immediate postnatal care visit? | 1. Yes<br>2. No | |
| 211 | Where you gave birth for index child? | 1. My home<br>2. Health Center<br>3. Hospital<br>4. In mom home | |
| 212 | How many times you gave birth? | _________________ | |
| **Part III: Knowledge related questions** | | | |
| 301 | At which time after delivery breastfeeding is initiated? | 1. Immediately after delivery<br>2. Within one hour after delivery<br>3. After one hour of delivery<br>4. After placenta is removed<br>5. Do not know | |
| 302 | At which time after delivery bathing of newborn is recommended? | 1. Immediately after delivery<br>2. After 24 hour of delivery<br>3. Before 24 hour of delivery<br>4. Do not know | |
| 303 | Do you know any neonatal danger signs? | 1. Yes<br>2. No | |
| 304 | How many neonatal danger signs do you now? | _______________________(in number) | |
| 305 | Mention neonatal danger signs? | 1. Poor sucking or not able to | |

| | | | |
|---|---|---|---|
| | *(Multiple response is possible or ask us Yes/No question)* | breastfeeding<br>2. Fever<br>3. omphalitis<br>4. Difficulty of breathing<br>5. Lethargic or unconscious<br>6. Hypothermia<br>7. Convulsion<br>8. Umbilical infection/such as redness of the cord<br>9. Yellowish discoloration of palms/soles<br>10. Vomiting | |
| **Part VI: Practice related questions** | | | |
| 401 | Which instrument was used to cut the cord? | 1. New blade<br>2. Used blade<br>3. Knife<br>4. Scissor<br>5. Other, Specify______________ | |
| 402 | Was the instrument used to cut cord boiled prior to use? | 1. Yes<br>2. No | |
| 403 | Which material was used to tie the cord? | 1. String or thread<br>2. Cord tie<br>3. Cord clamp<br>4. Other, Specify____________ | |
| 404 | Did anybody apply anything on the stump after the baby's cord was cut? | 1. Yes<br>2. No | If "No" skip to Q406 |
| 405 | What did he/she apply? | 1. Ointment/powder<br>2. Butter<br>3. Animal dung<br>4. Ash<br>5. Other(specify)__________________ ___________ | |
| 406 | How long after birth was your baby bathed for the first time? | 1. Immediately after delivery<br>2. After 24 hour of delivery<br>3. Before 24 hour of delivery | |
| 407 | Temperature of the water is? | 1. Warm<br>2. Cold | |
| 408 | Was your baby wiped off/dried within ten minute? | 1. Yes<br>2. No | |
| 409 | Was your baby wrapped in cloth or put on mother's body and covered with cloth before the placenta was delivered? | 1. Yes<br>2. No | If "No" skip to 411 |

| | | | |
|---|---|---|---|
| 410 | Which types of cloth you used to wrap the baby after bath? | 1. New cloth<br>2. Clean and dry old cloth<br>3. Soiled and old cloth<br>4. Other, Specify_________________ | |
| 411 | How long after birth did you first put the baby on the breast? | 1. Immediately after delivery<br>2. Within one hour after delivery<br>3. After one hour of delivery | |
| 412 | Did you give the first liquid (colostrum) that came out from your breasts? | 1. Yes<br>2. No | If “Yes” skip to 414 |
| 413 | What did you do with the first liquid (colostrum)? | ________________________________<br>___________ | |
| 414 | Did you give anything to drink other than breast milk in the first three days after birth? | 1. Yes<br>2. No | |
| 415 | If the above answer is “Yes”, which fluid you gave? | 1. Plain water<br>2. Butter<br>3. Animal Milk<br>4. Honey<br>5. Other, Specify___________ | |

**English Version Qualitative Guide**

**A Guide for qualitative part of the study which is supportive for quantitative part (focused group discussion guide)**

1. How you give essential newborn care for newborn?
2. What are different cultural/traditional practices/belief related to essential newborn care regarding the three domains (cord care, thermal care and breastfeeding after delivery)
3. Do you have any awareness/information regarding essential newborn care from different groups in your surrounding? What are those information’s?
